# Bis(imidazolium) galacta­rate dihydrate

**DOI:** 10.1107/S1600536810033532

**Published:** 2010-08-25

**Authors:** Graham Smith, Urs D. Wermuth

**Affiliations:** a Faculty of Science and Technology, Queensland University of Technology, GPO Box 2434, Brisbane, Queensland 4001, Australia

## Abstract

In the structure of the title salt, 2C_3_H_5_N_2_^+^·C_6_H_8_O_8_^2−^·2H_2_O, the galacta­rate dianions have crystallographic inversion symmetry and together with the water mol­ecules of solvation form hydrogen-bonded sheet substructures which extend along (110). The imidazolium cations link these sheets peripherally down *c* through carboxyl­ate O—H—N and N′—H⋯O_hy­droxy_ bridges, giving a three-dimensional framework structure.

## Related literature

For mention of mucic acid in the *Merck Index*, see: O’Neil (2001 ▶). For the structures of imidazolium hydrogen salts of aliphatic dicarb­oxy­lic acids, see: James & Matsushima (1976 ▶); MacDonald *et al.* (2001 ▶); Aakeröy & Hitchcock (1993 ▶); Fuller *et al.* (1995 ▶); Fukunaga & Ishida (2003 ▶); Trivedi *et al.* (2003 ▶). For the structures of galacta­ric acid, ammonium H galacta­rate, diammonium galacta­rate and copper(II) galacta­rate dihydrate, see: Jeffrey & Wood (1982 ▶), Bontchev & Moore (2005 ▶), Benetollo *et al.* (1993 ▶) and Ferrier *et al.* (1998 ▶) respectively. For graph-set analysis, see: Etter *et al.* (1990 ▶).
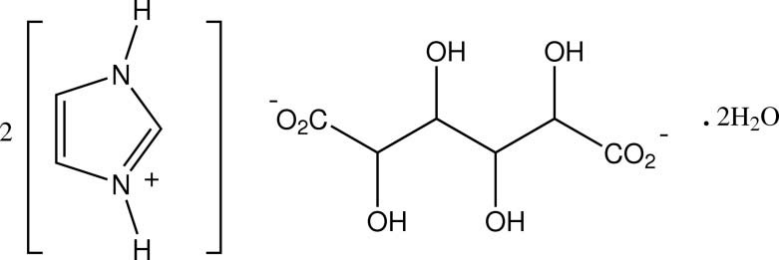


## Experimental

### 

#### Crystal data

2C_3_H_5_N_2_^+^·C_6_H_8_O_8_^2−^·2H_2_O
*M* *_r_* = 382.34
Triclinic, 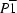

*a* = 6.9184 (4) Å
*b* = 7.1336 (4) Å
*c* = 9.3652 (5) Å
α = 92.000 (5)°
β = 100.559 (5)°
γ = 109.835 (6)°
*V* = 425.06 (5) Å^3^
*Z* = 1
Mo *K*α radiation
μ = 0.13 mm^−1^
*T* = 200 K
0.45 × 0.45 × 0.30 mm

#### Data collection

Oxford Diffraction Gemini-S CCD-detector diffractometer
Absorption correction: multi-scan (*CrysAlis PRO*; Oxford Diffraction, 2010 ▶) *T*_min_ = 0.965, *T*_max_ = 0.980
4949 measured reflections
1657 independent reflections
1431 reflections with *I* > 2σ(*I*)
*R*_int_ = 0.019

#### Refinement

*R*[*F* ^2^ > 2σ(*F* ^2^)] = 0.030
*wR*(*F* ^2^) = 0.082
*S* = 1.13
1657 reflections
142 parameters
H atoms treated by a mixture of independent and constrained refinement
Δρ_max_ = 0.30 e Å^−3^
Δρ_min_ = −0.20 e Å^−3^

### 

Data collection: *CrysAlis PRO* (Oxford Diffraction, 2010 ▶); cell refinement: *CrysAlis PRO*; data reduction: *CrysAlis PRO*; program(s) used to solve structure: *SIR92* (Altomare *et al.*, 1994 ▶); program(s) used to refine structure: *SHELXL97* (Sheldrick, 2008 ▶) within *WinGX* (Farrugia, 1999 ▶); molecular graphics: *PLATON* (Spek, 2009 ▶); software used to prepare material for publication: *PLATON*.

## Supplementary Material

Crystal structure: contains datablocks global, I. DOI: 10.1107/S1600536810033532/ng5021sup1.cif

Structure factors: contains datablocks I. DOI: 10.1107/S1600536810033532/ng5021Isup2.hkl

Additional supplementary materials:  crystallographic information; 3D view; checkCIF report

## Figures and Tables

**Table 1:** Hydrogen-bond geometry (Å, °)

*D*—H⋯*A*	*D*—H	H⋯*A*	*D*⋯*A*	*D*—H⋯*A*
N11—H11⋯O21	0.89 (2)	1.84 (2)	2.7311 (15)	175.9 (19)
N31—H31⋯O12^i^	0.890 (18)	1.795 (19)	2.6810 (14)	174 (2)
O21—H22⋯O1*W*	0.87 (2)	1.76 (2)	2.6324 (15)	177 (2)
O31—H32⋯O12^ii^	0.83 (2)	1.89 (2)	2.7104 (13)	170.9 (16)
O1*W*—H11*W*⋯O11^iii^	0.87 (3)	1.82 (3)	2.6799 (14)	170.9 (18)
O1*W*—H12*W*⋯O31^iv^	0.86 (3)	1.94 (3)	2.7763 (15)	164.4 (19)

Symmetry codes: (i) 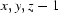; (ii) 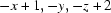; (iii) 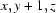; (iv) 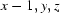.

**Fig. 1.**
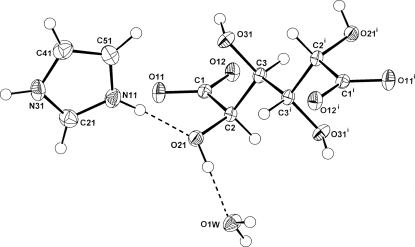
The molecular configuration and atom-numbering scheme for the cation, dianion and water species in (I). The galactarate dianion has inversion symmetry [symmetry code: (i) -x + 1, -y + 1, -z + 2]. Non-H atoms are shown as 50% probability ellipsoids and inter-species hydrogen bonds are shown as dashed lines.

**Fig. 2.**
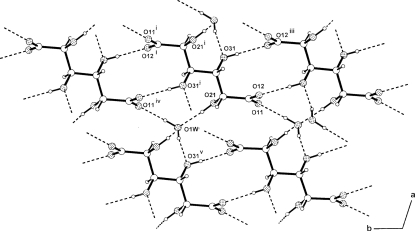
Hydrogen-bonded anion-water sheet substructures in (I), extending across (110) (imidazolium cations are omitted). For symmetry codes, see Table 1. Hydrogen bonds are shown as dashed lines.

**Fig. 3.**
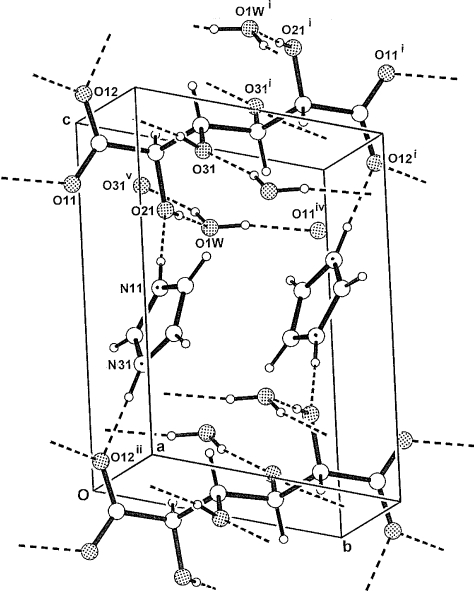
The three-dimensional structure of (I) viewed down the approximate a cell direction, showing the imidazolium bridges.

## supplementary crystallographic information

### Comment

Galactaric acid (mucic acid) (O'Neil, 2001) is the C6 homologue of tartaric acid but differs from it in being achiral and as well has only a small number of representative crystal structures in the CSD, *e.g.* the acid itself (Jeffrey & Wood, 1982), ammonium hydrogen galactarate (Bontchev & Moore, 2005), diammonium galactarate (Benetollo *et al.*, 1993) and some metal complexes, *e.g.* copper(II) galactarate dihydrate (a fungicide) (Ferrier *et al.*, 1998). Because the imidazolium cation has proved to be an excellent linking molecule for the generation of supramolecular layered structures particularly with dicarboxylic acids, including hydroxy acids (James & Matsushima, 1976; MacDonald *et al.*, 2001; Aakeröy & Hitchcock, 1993; Fuller *et al.*, 1995; Fukunaga & Ishida, 2003; Trivedi *et al.*, 2003), we carried out a 1:2 stoichiometric reaction of galactaric acid with imidazole in aqueous ethanol and obtained large relatively hard, chemically stable crystals of the title compound, 2(CH_6_N_3_^+^) C_6_H_8_O_8_^2-^. 2H_2_O (I), and the structure is reported here.

In the structure of (I) (Fig. 1), the galactarate anions lie across crystallographic inversion centres which is also the case in the structure of the parent acid (Jeffrey & Wood, 1982). Hydrogen-bonded anion-water sheets extending across the <100> planes in the unit cell (Fig. 2) are formed through hydroxyl O31–*H*···O12^iii^_carboxyl_ and water-bridging O31···O11^iv^_carboxyl_ interactions (for symmetry codes, see Table 1). These include *R*^2^_2_(12) and *R*^3^_3_(12) cyclic motifs (Etter *et al.*, 1990). The layered substructures are linked peripherally down the *c* cell direction by the imidazolium cations through carboxyl *O*···*H*—*N*,*N*'—*H*···*O*`_hydroxyl_ bridges giving a three-dimensional framework structure (Fig. 3). The structure of (I) differs from those of the anhydrous 1:1 salts of the hydrogen dicarboxylates (MacDonald *et al.*, 2001) in which the bridging imidazolium cations are incorporated within two-dimensional layered structures.

### Experimental

The title compound was synthesized by heating together under reflux for 10 minutes 1 mmol of galactaric acid (mucic acid) and 2 mmol of imidazole in 50 ml of 50% ethanol-water. After concentration to *ca* 30 ml, partial room temperature evaporation of the hot-filtered solution gave large colourless plates of (I) (m.p. 435 K) from which a suitable analytical specimen was cleaved.

### Refinement

Hydrogen atoms potentially involved in hydrogen-bonding interactions were located by difference methods and their positional and isotropic displacement parameters were refined. Other H atoms were included in the refinement in calculated positions (C–H_aromatic_ = 0.95 Å and others = 1.00 Å) and allowed to ride, with *U*_iso_(H) = 1.2*U*_eq_(C).

### Crystal data

**Table:**

2C_3_H_5_N_2_^+^·C_6_H_8_O_8_^2^^−^·2H_2_O	*Z* = 1
*M**_r_* = 382.34	*F*(000) = 202
Triclinic, *P*1	*D*_x_ = 1.494 Mg m^−^^3^
Hall symbol: -P 1	Melting point: 435 K
*a* = 6.9184 (4) Å	Mo *K*α radiation, λ = 0.71073 Å
*b* = 7.1336 (4) Å	Cell parameters from 3387 reflections
*c* = 9.3652 (5) Å	θ = 3.5–28.7°
α = 92.000 (5)°	µ = 0.13 mm^−^^1^
β = 100.559 (5)°	*T* = 200 K
γ = 109.835 (6)°	Plate, colourless
*V* = 425.06 (5) Å^3^	0.45 × 0.45 × 0.30 mm

### Data collection

**Table:**

Oxford Diffraction Gemini-S CCD-detector diffractometer	1657 independent reflections
Radiation source: Enhance (Mo) X-ray source	1431 reflections with *I* > 2σ(*I*)
graphite	*R*_int_ = 0.019
ω scans	θ_max_ = 26.0°, θ_min_ = 3.5°
Absorption correction: multi-scan (*CrysAlis PRO*; Oxford Diffraction, 2010)	*h* = −8→8
*T*_min_ = 0.965, *T*_max_ = 0.980	*k* = −8→8
4949 measured reflections	*l* = −11→11

### Refinement

**Table:**

Refinement on *F*^2^	Primary atom site location: structure-invariant direct methods
Least-squares matrix: full	Secondary atom site location: difference Fourier map
*R*[*F*^2^ > 2σ(*F*^2^)] = 0.030	Hydrogen site location: inferred from neighbouring sites
*wR*(*F*^2^) = 0.082	H atoms treated by a mixture of independent and constrained refinement
*S* = 1.13	*w* = 1/[σ^2^(*F*_o_^2^) + (0.0444*P*)^2^ + 0.0516*P*] where *P* = (*F*_o_^2^ + 2*F*_c_^2^)/3
1657 reflections	(Δ/σ)_max_ = 0.001
142 parameters	Δρ_max_ = 0.30 e Å^−^^3^
0 restraints	Δρ_min_ = −0.20 e Å^−^^3^

### Special details

**Table:**

Geometry. Bond distances, angles *etc*. have been calculated using the rounded fractional coordinates. All su's are estimated from the variances of the (full) variance-covariance matrix. The cell e.s.d.'s are taken into account in the estimation of distances, angles and torsion angles
Refinement. Refinement of *F*^2^ against ALL reflections. The weighted *R*-factor *wR* and goodness of fit *S* are based on *F*^2^, conventional *R*-factors *R* are based on *F*, with *F* set to zero for negative *F*^2^. The threshold expression of *F*^2^ > σ(*F*^2^) is used only for calculating *R*-factors(gt) *etc*. and is not relevant to the choice of reflections for refinement. *R*-factors based on *F*^2^ are statistically about twice as large as those based on *F*, and *R*- factors based on ALL data will be even larger.

### Fractional atomic coordinates and isotropic or equivalent isotropic displacement parameters (Å^2^)

**Table:**

	*x*	*y*	*z*	*U*_iso_*/*U*_eq_	
O11	0.18384 (15)	−0.07229 (13)	0.80626 (9)	0.0234 (3)	
O12	0.26773 (14)	−0.02982 (13)	1.04961 (9)	0.0207 (3)	
O21	0.19607 (14)	0.29951 (14)	0.78260 (9)	0.0203 (3)	
O31	0.62559 (14)	0.35914 (13)	0.90945 (10)	0.0197 (3)	
C1	0.23436 (18)	0.03283 (17)	0.92558 (13)	0.0154 (3)	
C2	0.25848 (18)	0.25478 (17)	0.92684 (12)	0.0151 (3)	
C3	0.48566 (18)	0.38863 (17)	0.99549 (13)	0.0153 (3)	
N11	0.34766 (19)	0.22454 (18)	0.54570 (12)	0.0261 (4)	
N31	0.35478 (19)	0.13600 (18)	0.32587 (12)	0.0265 (4)	
C21	0.2302 (2)	0.1404 (2)	0.41585 (14)	0.0269 (4)	
C41	0.5588 (2)	0.2202 (2)	0.40018 (15)	0.0302 (5)	
C51	0.5544 (2)	0.2764 (2)	0.53780 (15)	0.0285 (4)	
O1W	−0.02769 (16)	0.53271 (15)	0.78655 (11)	0.0244 (3)	
H22	0.118 (3)	0.373 (3)	0.7852 (19)	0.046 (5)*	
H2	0.16380	0.28050	0.98780	0.0180*	
H3	0.52450	0.35200	1.09610	0.0180*	
H32	0.657 (3)	0.260 (3)	0.9315 (18)	0.040 (5)*	
H11	0.301 (3)	0.245 (3)	0.625 (2)	0.048 (5)*	
H21	0.08100	0.09150	0.39160	0.0320*	
H31	0.318 (3)	0.084 (3)	0.233 (2)	0.044 (5)*	
H41	0.68070	0.23610	0.36180	0.0360*	
H51	0.67240	0.34010	0.61480	0.0340*	
H11W	0.043 (3)	0.660 (4)	0.803 (2)	0.057 (6)*	
H12W	−0.127 (4)	0.500 (3)	0.835 (2)	0.059 (6)*	

### Atomic displacement parameters (Å^2^)

**Table:**

	*U*^11^	*U*^22^	*U*^33^	*U*^12^	*U*^13^	*U*^23^
O11	0.0320 (5)	0.0168 (5)	0.0185 (5)	0.0058 (4)	0.0047 (4)	−0.0032 (4)
O12	0.0290 (5)	0.0158 (5)	0.0174 (4)	0.0077 (4)	0.0056 (4)	0.0024 (3)
O21	0.0257 (5)	0.0230 (5)	0.0157 (5)	0.0135 (4)	0.0032 (4)	0.0023 (4)
O31	0.0214 (5)	0.0149 (5)	0.0265 (5)	0.0079 (4)	0.0113 (4)	0.0032 (4)
C1	0.0129 (6)	0.0148 (6)	0.0174 (6)	0.0026 (5)	0.0051 (4)	0.0001 (5)
C2	0.0185 (6)	0.0143 (6)	0.0132 (6)	0.0058 (5)	0.0049 (5)	0.0009 (5)
C3	0.0185 (6)	0.0136 (6)	0.0144 (6)	0.0052 (5)	0.0054 (5)	0.0017 (5)
N11	0.0347 (7)	0.0308 (7)	0.0175 (6)	0.0160 (5)	0.0084 (5)	0.0031 (5)
N31	0.0379 (7)	0.0260 (6)	0.0152 (6)	0.0116 (5)	0.0044 (5)	0.0010 (5)
C21	0.0273 (7)	0.0304 (8)	0.0230 (7)	0.0104 (6)	0.0043 (6)	0.0070 (6)
C41	0.0311 (8)	0.0336 (8)	0.0301 (8)	0.0134 (6)	0.0120 (6)	0.0064 (6)
C51	0.0286 (7)	0.0294 (8)	0.0241 (7)	0.0085 (6)	0.0007 (6)	0.0004 (6)
O1W	0.0231 (5)	0.0180 (5)	0.0325 (6)	0.0063 (4)	0.0095 (4)	−0.0005 (4)

### Geometric parameters (Å, °)

**Table:**

O11—C1	1.2465 (15)	N11—H11	0.89 (2)
O12—C1	1.2690 (15)	N31—H31	0.890 (18)
O21—C2	1.4223 (14)	C1—C2	1.5341 (16)
O31—C3	1.4293 (16)	C2—C3	1.5375 (18)
O21—H22	0.87 (2)	C3—C3^i^	1.5303 (16)
O31—H32	0.83 (2)	C2—H2	1.0000
O1W—H11W	0.87 (3)	C3—H3	1.0000
O1W—H12W	0.86 (3)	C41—C51	1.345 (2)
N11—C21	1.3249 (17)	C21—H21	0.9500
N11—C51	1.367 (2)	C41—H41	0.9500
N31—C21	1.3178 (19)	C51—H51	0.9500
N31—C41	1.369 (2)		
			
C2—O21—H22	108.6 (11)	O31—C3—C3^i^	107.52 (10)
C3—O31—H32	110.6 (13)	O21—C2—H2	108.00
H11W—O1W—H12W	111 (2)	C3—C2—H2	108.00
C21—N11—C51	108.65 (12)	C1—C2—H2	108.00
C21—N31—C41	108.66 (11)	O31—C3—H3	109.00
C51—N11—H11	125.3 (14)	C2—C3—H3	109.00
C21—N11—H11	126.1 (14)	C3^i^—C3—H3	109.00
C41—N31—H31	123.6 (14)	N11—C21—N31	108.63 (13)
C21—N31—H31	127.7 (14)	N31—C41—C51	107.15 (13)
O12—C1—C2	116.02 (10)	N11—C51—C41	106.92 (12)
O11—C1—O12	124.82 (11)	N31—C21—H21	126.00
O11—C1—C2	119.16 (10)	N11—C21—H21	126.00
O21—C2—C3	111.32 (10)	N31—C41—H41	126.00
O21—C2—C1	110.05 (9)	C51—C41—H41	126.00
C1—C2—C3	110.45 (10)	N11—C51—H51	127.00
O31—C3—C2	109.98 (9)	C41—C51—H51	127.00
C2—C3—C3^i^	112.11 (10)		
			
C21—N11—C51—C41	−0.42 (16)	C1—C2—C3—C3^i^	−177.68 (10)
C51—N11—C21—N31	0.31 (16)	C1—C2—C3—O31	62.76 (12)
C21—N31—C41—C51	−0.19 (16)	O21—C2—C3—C3^i^	59.76 (13)
C41—N31—C21—N11	−0.08 (16)	O31—C3—C3^i^—O31^i^	179.98 (12)
O11—C1—C2—O21	5.67 (17)	C2—C3—C3^i^—O31^i^	59.01 (12)
O12—C1—C2—O21	−174.06 (11)	C2—C3—C3^i^—C2^i^	−179.98 (12)
O12—C1—C2—C3	62.63 (14)	O31—C3—C3^i^—C2^i^	−59.01 (12)
O11—C1—C2—C3	−117.63 (13)	N31—C41—C51—N11	0.36 (16)
O21—C2—C3—O31	−59.81 (13)		

Symmetry codes: (i) −*x*+1, −*y*+1, −*z*+2.

### Hydrogen-bond geometry (Å, °)

**Table:**

*D*—H···*A*	*D*—H	H···*A*	*D*···*A*	*D*—H···*A*
N11—H11···O21	0.89 (2)	1.84 (2)	2.7311 (15)	175.9 (19)
N31—H31···O12^ii^	0.890 (18)	1.795 (19)	2.6810 (14)	174 (2)
O21—H22···O1W	0.87 (2)	1.76 (2)	2.6324 (15)	177 (2)
O31—H32···O12^iii^	0.83 (2)	1.89 (2)	2.7104 (13)	170.9 (16)
O1W—H11W···O11^iv^	0.87 (3)	1.82 (3)	2.6799 (14)	170.9 (18)
O1W—H12W···O31^v^	0.86 (3)	1.94 (3)	2.7763 (15)	164.4 (19)
C21—H21···O11^vi^	0.95	2.32	3.0935 (17)	138
C41—H41···O11^vii^	0.95	2.42	3.2273 (18)	142
C51—H51···O1W^viii^	0.95	2.34	3.2827 (18)	173

Symmetry codes: (ii) *x*, *y*, *z*−1; (iii) −*x*+1, −*y*, −*z*+2; (iv) *x*, *y*+1, *z*; (v) *x*−1, *y*, *z*; (vi) −*x*, −*y*, −*z*+1; (vii) −*x*+1, −*y*, −*z*+1; (viii) *x*+1, *y*, *z*.
